# Additional Value of Machine-Learning Computed Tomographic Angiography-Based Fractional Flow Reserve Compared to Standard Computed Tomographic Angiography

**DOI:** 10.3390/jcm9030676

**Published:** 2020-03-03

**Authors:** Dirk Lossnitzer, Leonard Chandra, Marlon Rutsch, Tobias Becher, Daniel Overhoff, Sonja Janssen, Christel Weiss, Martin Borggrefe, Ibrahim Akin, Stefan Pfleger, Stefan Baumann

**Affiliations:** 1 First Department of Medicine-Cardiology, University Medical Centre Mannheim, Mannheim, Germany, DZHK (German Centre for Cardiovascular Research), partner site Heidelberg/Mannheim, Mannheim, Germany and ECAS (European Center for Angioscience), Faculty of Medicine Mannheim, Heidelberg University, 68167 Mannheim, Germany; L.Chandra@stud.uni-heidelberg.de (L.C.); rutsch@stud.uni-heidelberg.de (M.R.); tobias.becher@umm.de (T.B.); martin.borggrefe@umm.de (M.B.); ibrahim.akin@umm.de (I.A.); pfleger@kardangma.de (S.P.); stefan.Baumann@umm.de (S.B.); 2 Institute of Clinical Radiology and Nuclear Medicine, University Medical Center Mannheim, Faculty of Medicine Mannheim, Heidelberg University, 68167 Mannheim, Germany; danieloverhoff@web.de (D.O.); Sonja.Janssen@umm.de (S.J.); 3 Medical Faculty Mannheim, Department of Medical Statistics and Biomathematics, University Medical Center Mannheim, Heidelberg University, 68167 Mannheim, Germany; christel.Weiss@umm.de

**Keywords:** atherosclerosis, coronary artery disease, coronary physiology, coronary CT angiography, fractional flow reserve, CT derived fractional flow reserve, non-invasive test, revascularization

## Abstract

**Background:** Machine-learning-based computed-tomography-derived fractional flow reserve (CT-FFR_ML_) obtains a hemodynamic index in coronary arteries. We examined whether it could reduce the number of invasive coronary angiographies (ICA) showing no obstructive lesions. We further compared CT-FFR_ML_-derived measurements to clinical and CT-derived scores. **Methods:** We retrospectively selected 88 patients (63 ± 11years, 74% male) with chronic coronary syndrome (CCS) who underwent clinically indicated coronary computed tomography angiography (cCTA) and ICA. cCTA image data were processed with an on-site prototype CT-FFR_ML_ software. **Results:** CT-FFR_ML_ revealed an index of >0.80 in coronary vessels of 48 (55%) patients. This finding was corroborated in 45 (94%) patients by ICA, yet three (6%) received revascularization. In patients with an index ≤ 0.80, three (8%) of 40 were identified as false positive. A total of 48 (55%) patients could have been retained from ICA. CT-FFR_ML_ (AUC = 0.96, *p* ≤ 0.0001) demonstrated a higher diagnostic accuracy compared to the pretest probability or CT-derived scores and showed an excellent sensitivity (93%), specificity (94%), positive predictive value (PPV; 93%) and negative predictive value (NPV; 94%). **Conclusion:** CT-FFR_ML_ could be beneficial for clinical practice, as it may identify patients with CAD without hemodynamical significant stenosis, and may thus reduce the rate of ICA without necessity for coronary intervention.

## 1. Introduction

In the last decade, coronary computed tomography angiography (cCTA) has been primarily used as a non-invasive method to obtain morphological information about the status of atherosclerosis and plaque in coronary arteries [1]. Studies including PROMISE [2] and SCOT-HEART [3] examined the diagnostic yield of an initial cCTA scan compared to standard non-invasive functional testing such as exercise electrocardiography, stress echocardiography, cardiac Magnetic Resonance Imaging (MRI) or nuclear stress testing in symptomatic patients suspected of suffering from obstructive coronary artery disease (CAD). The high negative predictive value of cCTA, specifically in patients with a lower pre-test probability for CAD, allows cardiologists to rule out obstructive CAD with high certainty and thus reduces the number of concomitant unnecessary invasive procedures [4,5]. One major drawback of cCTA for the detection of obstructive CAD and usage as gatekeeper for ICA is the low specificity. cCTA frequently overrates the severity of coronary stenosis in heavily calcified coronary arteries, resulting in false positive results that trigger a referral to an interventional cardiologist [2,3,6,7].

The current gold standards for the assessment of the hemodynamic significance of a coronary lesion are pressure-derived indices including the invasively measured fractional flow reserve (FFR) [6] and, just recently, the instantaneous wave-free ratio (iwFR) [8]. In patients with chronic coronary syndrome (CCS), FFR-guided percutaneous coronary intervention (PCI) is superior regarding morbidity and prognostic outcome compared to an optimal medical therapy (OMT) alone [9,10]. FFR measures the pressure proximal and distal to a lesion under maximal hyperemia induced by the vasodilator adenosine and calculates the ratio between these two values. In the last two decades, FFR guided revascularization has been established in clinical practice. Prominently, the DEFER study demonstrated that patients do not benefit from the treatment of coronary lesions that do not cause myocardial ischemia as determined by FFR compared to OMT [11]. Furthermore, the FAME study reported a decrease in major adverse cardiac events (MACE) when treating intermediate coronary lesions guided by an FFR Index-value of ≤ 0.80 only [12]. SWEDEHEART and DEFINE FLAIR finally demonstrated that revascularization guided by iwFR yielded comparable results to FFR [13,14]. Different from FFR, iwFR is obtained under resting conditions in a wave-free period during the diastole of the cardiac cycle. However, the results of the large multicenter ISCHEMIA trial, which were recently presented, indicate that stable patients with moderate ischemia did not profit from a routine invasive therapy [15,16]. Due to inconclusive outcome data, an extended long-term follow-up is expected. Although it is not yet published, the study shows even more the importance of a precise pre-invasive selection of patients [15,16].

CT-derived fractional flow reserve (CT-FFR; FFR computed from resting cCTA images) appears to have a high correlation with invasive FFR [7]. CT-FFR is a promising novel approach that allows both anatomical and functional assessment of a coronary stenoses and has an improved specificity compared to a cCTA-only approach. The prospective multicenter PLATFORM trial already demonstrated a lower rate of patients showing no obstructive coronary lesions with ICA, when CT-FFR was obtained previously as a gatekeeper [17,18]. As an additional advantage, CT-FFR does not require additional radiation or pharmaceutical stress agents [19]. A recent improvement in the development of CT-FFR is the introduction of an on-site prototype software based on a machine-learning algorithm (CT-FFR_ML_). CT-FFR_ML_ provides a significant reduction in the required computation time compared to CT-FFR based on computational fluid dynamics [20]. CT-FFR_ML_ has already been successfully validated against both invasive FFR and conventional off-site CT-FFR [7,20]. The overall goal of coronary diagnostics is the accurate characterization of lesion location, morphology and hemodynamic impact in light of patient characteristics and pre-test probability, with the ultimate goal to derive treatment options and risk stratification. Therefore, several scores have been developed condensing patients’ data. The CAD consortium basic pretest probability predicts the chance of having an obstructive CAD and was recommended by the ESC guidelines in 2013 [6] and 2019 [4]. The Agatston score [21] quantifies the amount of deposited calcium to predict coronary events. A newly developed comprehensive cCTA score, which is based on the location and the severity of the lesion as well as the plaque composition may also be used to characterize CAD severity [22].

The purpose of the present study was to examine the potential impact of the new machine-learning-based CT-FFR_ML_ prototype on clinical practice, particularly the diagnostic and therapeutic pathway of patients with suspected CAD. We were further interested in examining whether application of CT-FFR_ML_ could reduce the number of patients who undergo ICA following cCTA with the diagnosis of non-obstructive CAD.

## 2. Materials and Methods

### 2.1. Patient Population

This study is an observational, retrospective, single-center study. The population consisted of 88 consecutive patients treated between January 2013 and December 2018, in which a clinically indicated cCTA scan revealed a diameter stenosis of >50% in one or more coronary arteries and who received an ICA within 3 months thereafter. We did not include patients selected by other non-invasive tests. Patients without CAD or a diameter stenosis <50% on cCTA, non-diagnostic CT image quality, previous stent implantation in the vessel of interest, prior coronary artery bypass grafting (CABG), subtotal or chronic total occlusions or coronary anomalies were excluded. Patients suspected of CCS with a positive cCTA (>50% diameter stenosis) received treatment according to current guidelines [4,6]. This implicated ICA and subsequent revascularization, or OMT, depending on stenosis severity, with the final treatment decision at the discretion of the treating cardiologist. Baseline characteristics, cardiovascular risk factors and blood values were obtained from medical records. The CAD consortium basic score was used to determine pretest probability for CAD according to the European society of cardiology (ESC) guidelines [23]. 

The present study was approved by the local Institutional Review Board (IRB number 2019-825R) with a waiver of informed consent due to its retrospective design and was conducted in accordance with the Declaration of Helsinki. 

### 2.2. Risk-Stratification

The CAD consortium basic pretest probability score uses age, sex and symptom quality to calculate CAD probability [23,24]. The Agatston score quantifies the calcium status in the coronary artery tree, a surrogate for CAD severity, and is calculated with the Agatston method [25]. The newly developed comprehensive cCTA score, a recently published risk-stratification score based on cCTA-imaging parameters including the location (17-segment model), the severity (diameter stenosis of <50% or ≥50%) and the plaque composition (calcified, non-calcified, mixed) of the lesion, was also used to assess CAD severity [22].

### 2.3. cCTA Acquisition and Analysis 

Acquisition and analysis of cCTA images were performed according to local scan protocols and as previously described in detail [20]. All cCTAs were performed using either a 2x128-slice dual-source CT system (Somatom Definition Flash; Siemens Healthineers, Forchheim, Germany) or a 2x192-slice dual-source CT System (Somatom FORCE, Siemens Healthineers, Forchheim, Germany). The majority of patients (*n* = 78, 89%) received an initial non-contrast scan to assess the coronary calcium score with a dedicated software application (CaScore; Siemens Healthineers, Forchheim, Germany) [25]. Thereafter, all patients underwent electrocardiographic-gated contrast-enhanced cCTA acquisition. Sublingual nitroglycerin and i.v. beta-blocker were administered if there were no contraindications. Iodinated contrast agent (Imeron 400; Bracco Imaging S.p.A., Milan, Italy) adapted to the weight of each patient was injected in an antecubital vein using a power injector (Stellant D; Medrad, Warrendale, PA, USA) followed by a 20 mL saline chaser at a rate of 5 mL/s. CCTA images were analyzed on a commercially available multi-modality 3D-enabled workstation (Syngo VE36A; Siemens Healthineers, Forchheim, Germany) by two experienced cardiovascular radiologists. The diameter stenosis were graded as mild (25–49% diameter stenosis), moderate (50–69% diameter stenosis), severe (70–99% diameter stenosis), or occluded (100% diameter stenosis) with location based on an 18-segment coronary artery model [26]. Two experienced cardiologists specialized in cardiac CT imaging independently evaluated and ranked the image quality of all cCTA-scans using a 5-point Likert scale (1 = non-diagnostic; 2 = diagnostic despite impairment by image noise, artifacts, and/or low contrast opacification; 3 = moderate image noise with sufficient intraluminal visibility, artifacts may be present; 4 = good vessel contrast in the absence of major artifacts, low image noise; 5 = excellent, no diagnostic limitations).

### 2.4. Analysis of Computed Tomography Angiography based Fractional Flow Reserve 

**Figure 1:**
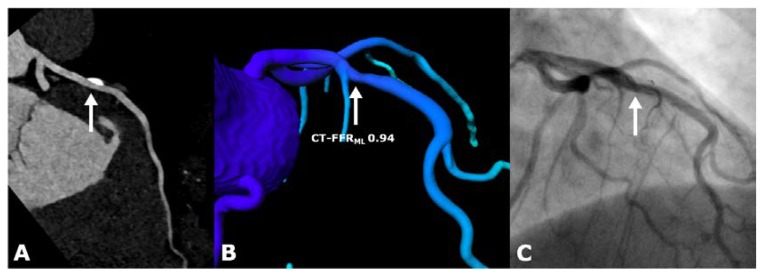
53 year old female patient with suspected coronary artery disease (CAD) and a history of smoking. (**A**) Calcified plaque of the mid left anterior descending artery (LAD) in a sectional image by coronary computed tomography angiography (cCTA) (arrow). (**B**) The computed-tomography-derived fractional flow reserve (CT-FFR_ML_) software (cFFR, version 3.1, Siemens Healthineers, Forchheim, Germany) illustrates a 3D-model of the mid LAD with a not-flow-limiting stenosis and a CT-FFR_ML_ value of 0.94 (arrow). (**C**) Invasive coronary angiography shows a mild stenosis in the mid LAD (arrow).

**Figure 2:**
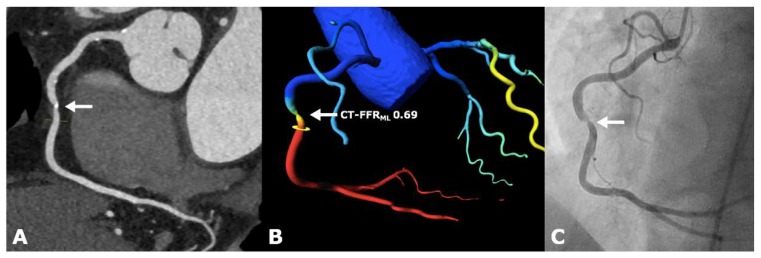
66 year old female patient with suspected progress of CAD and a high cardiovascular risk profile. (**A**) Severe stenosis (>70% diameter stenosis) of the mid-right coronary artery (RCA) in a sectional image by cCTA (arrow). (**B**) The CT-FFR_ML_ software (cFFR, version 3.1, Siemens Healthineers, Forchheim, Germany) illustrates a 3D-model of the mid RCA with a hemodynamically significant stenosis and a CT-FFR_ML_ value of 0.69 (arrow). (**C**) Invasive coronary angiography shows a significant stenosis in the mid RCA (arrow).

cCTA datasets of this study population were used retrospectively and a CT-FFR_ML_ value for each coronary vessel was computed in all patients. The CT-FFR_ML_ values were generated on-site on a conventional workstation (Syngo VE36A; Siemens Healthineers, Forchheim, Germany) based on the original cCTA images without the need for the further acquisition of data, radiation or additional use of pharmacological stress agents such as adenosine. In our study, we used a research software prototype (Siemens Healthineers cFFR, Forchheim, Germany, version 3.1), which is currently not commercially available. The software applied the following semiautomatic approach: centerlines of the coronary arteries were computed automatically with the option of manual adjustment. The vessel contours were proposed by the system and alignment was corrected manually if necessary. From this setting, a 3-dimensional anatomic model of the aortic root and epicardial coronary vessels with a diameter greater than 2 mm was calculated, which served as a starting point for the algorithm. The software used in our study is based on a machine learning algorithm that is based on an artificial intelligence using a deep learning framework. It was initially trained by using a large, synthetically created database of generated coronary artery trees and was later independently validated against an algorithm that is based on computational fluid dynamics [20]. Therefore, it learned “the complex relationship between the anatomy of the coronary tree and its corresponding hemodynamics” [20]. Calculations of the machine learning algorithm are further based on 28 patients’ specific weighted features, like the coronary morphology and their interactions [20]. The computational fluid dynamics algorithm computes coronary blood flow during a hyperemic state, depending on the geometric coronary artery model as well as heart models that reflect the inlet of the aorta and the microcirculation based on patient-specific rest state conditions including systolic and diastolic blood pressure, heart rate and ventricular mass [20]. It further uses a hybrid reduced-order for fast flow computation, which allows a more time-efficient and on-site measurement on a regular workstation. The physical law behind computational fluid dynamics is the Navier–Stokes equation [20]. Both algorithms, CT-FFR by computational fluid dynamics (CT-FFR_CFD_) and machine learning (CT-FFR_ML)_, generate a value for every point of the coronary artery tree, using the ratio of the average aortic and local pressure over a cardiac cycle. Thus, a 3-dimensional color-coded mesh of the coronary artery tree is created in combination with functional information at each segment of interest [7]. In order to analyze the clinical impact in our retrospective study design, CT-FFR_ML_ was virtually placed between the cCTA scan and the already realized ICA. The result of the analysis, a CT-FFR_ML_ value above or below the cut-off value of 0.80 (Figure 1 and Figure 2), guided the hypothetical downstream treatment. On a per-patient analysis, the lesion that appeared hemodynamically significant on cCTA was used as a measurement point for CT-FFR_ML_ (vessel of interest). Next, we compared this modified hypothetical diagnostic strategy to the standard procedure to see if integrating CT-FFR_ML_ into the diagnostic workflow could have prevented patients from invasive diagnostics. 

### 2.5. Coronary Angiography

Invasive coronary angiography was conducted corresponding to current guidelines of the ESC [4,6,8]. Each coronary vessel initially underwent visual evaluation. The stenoses were ranked from 0 to 4 according to AHA-guidelines [27]. If the hemodynamic relevance of a coronary stenosis was not apparent by visual assessment, FFR- or iwFR-measurements were used according to the current guidelines [12]. This pressure-derived index was obtained by using a floppy-tipped guide wire with a pressure-sensing transducer (Verrata Plus, Volcano Corporation Koninklijke Philips N.V., Amsterdam, The Netherlands), which was advanced over the segment of the culprit lesion. A FFR-Index of ≤0.80 and an iwFR-Index of ≤0.89 were adopted as cut-off values for hemodynamic relevance, as established in previous studies [28]. 

### 2.6. Statistical Analysis

All analyses were performed using SAS (release 9.4, SAS Institute Inc., Cary, NC, USA). Categorical variables are presented by absolute and relative frequencies. Continuous variables are presented either as mean ± standard deviation (SD) and are evaluated with the Mann–Whitney U Test. Furthermore, logistic regression analyses have been performed and ROC curves have been created to evaluate the scores as well as the CT-FFR_ML_ Index. To assess the inter-rater-agreement, a weighted Kappa coefficient was calculated according to Cohen and Fleiss. A test-value with *p* < 0.05 was considered statistically significant.

## 3. Results

### 3.1. Demographics and Study Population

**Figure 3:**
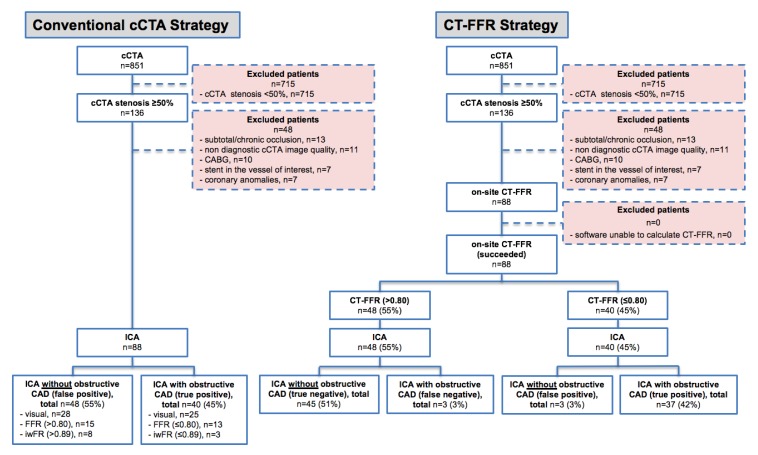
Study Flow-Chart. CT-FFR = CT-derived fractional flow reserve, cCTA = coronary computed tomography angiography, ICA = invasive coronary angiography, CAD = coronary artery disease, CABG = coronary artery bypass graft.

In our single-center study, we initially screened 851 patients who had an indication for a cCTA scan on the grounds of suspected de-novo or progressive CAD between January 2013 and December 2018. A total of 715 patients demonstrated CAD with a coronary lesion of <50% in diameter stenosis and were thus excluded. Thirteen (10%) out of 136 patients with subtotal or chronic total occlusion, seven (5%) patients with coronary anomalies, seven patients (5%) with prior stenting and another 10 (7%) patients after CABG were excluded. Visual evaluation of cCTA data of 11 (8%) patients was not possible due to insufficient image quality (Figure 3). Thus, in 88 cases (74% male, average age 63 ± 11 y) with a coronary diameter stenosis ≥50%, CT-FFR_ML_ was conducted. The mean pre-test probability for having CAD was 25% ± 21%. 69 (78%) had hypertension, 34 (39%) were smokers, 13 (15%) had diabetes, 39 (44%) had dyslipidemia and 19 (22%) had a positive family history of CAD. The mean number of cardiovascular risk factors was 2.0 ± 0.9. A total of 62 (70%) patients took at least one, and 45 (51%) at least two of the following medications: aspirin, P2Y_12_ inhibitor, statin, beta-blocker, calcium channel blocker (CCB), angiotensin-converting-enzyme inhibitor or AT_1_ receptor blocker (ARB) or nitrates. Baseline characteristics of all included patients are presented in Table 1. 

### 3.2. Risk Stratification

**Figure 4:**
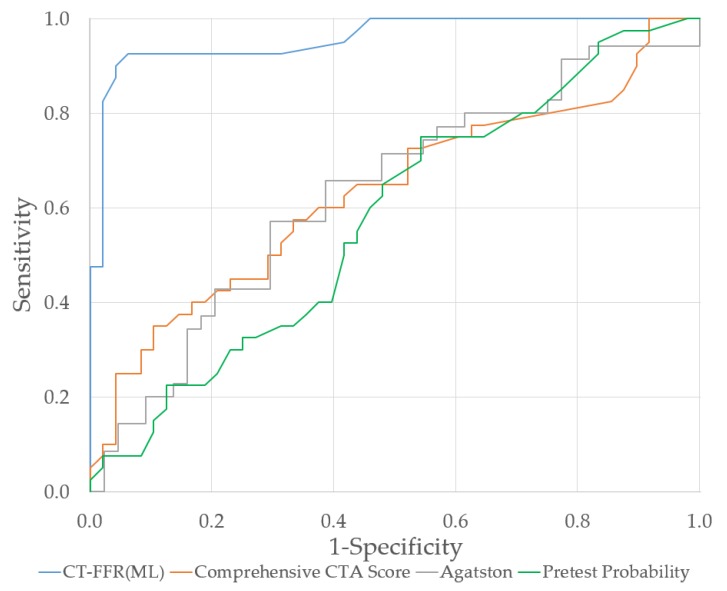
ROC-curve of pretest probability, Agatston score, the comprehensive CTA score and CT-FFR_ML_.

The Agatston score as well as the comprehensive CTA score differed significantly between the revascularized and not-revascularized group (*p* = 0.0467, *p* = 0.0324) (Table 2). Logistic regression analysis revealed that neither the pretest-probability (AUC = 0.578; *p* = 0.3212), the Agatston score (AUC = 0.631; *p* = 0.1448) nor the comprehensive CTA score (AUC = 0.633; *p* = 0.0158) demonstrated a sufficient diagnostic accuracy regarding revascularization (Figure 4). In view of the Agatston score, the average value of the vessel of interest (≥50% diameter stenosis) was 218 ± 257. The mean ratio of the Agatston score of the vessel of interest and the total score was 0.58. In our population, the newly developed comprehensive cCTA score achieved a mean value of 9.7 ± 5.5. Fourteen patients had a score less than 5, 68 between 5 and 20, and 6 ≥ 20.

### 3.3. cCTA and CT-FFR

Due to the study design, all 88 (100%) patients had at least one diameter stenosis of ≥50% in cCTA. On a per vessel analysis, cCTA observed 137 (52%) coronary arteries with a diameter stenosis ≥50% from a total of 264 vessels. The degree of the stenosis was more often graded as moderate or severe in the left anterior descending (LAD) [74 (54%)] than in the right coronary artery (RCA) [33 (24%)] or the left circumflex (RCX) [27 (20%)]. In the group of patients with ≥70% diameter stenosis, the CT-FFR_ML_ value (normal value >0.80) on a per-lesion analysis was generally lower (mean: 0.72) than those with a 50–69% diameter stenosis (mean: 0.80) lesion as assessed by cCTA. The interobserver variability for the evaluation of image quality by the two cardiologists showed a moderately good agreement with a weighted kappa of 0.64 (95% confidence interval, 0.52 to 0.76) (Table 3).

### 3.4. CT-FFR Analysis and Reclassification 

The CT-FFR_ML_ analysis of the vessels of 48 (55%) patients revealed a normal index of >0.80 in at least one stenosis. A total of 45 (94%) patients showed no obstruction in ICA and were therefore classified correctly. Three (6%) patients showed an obstruction in ICA despite a CT-FFR_ML_ value of >0.80, and were thus classified as a false negative. In the group of patients with a CT-FFR_ML_ values of ≤0.80 (40, 45%), 37 (93%) were revascularized in ICA. Hypothetically, three (7%) patients without any obstruction would have received a diagnostic ICA without undergoing revascularization (Table 4). The procedural time for the CT-FFR_ML_ analysis includes the semiautomatic process of computing the centerline and the contours of the coronary artery lumen, as well as their subsequent manual adjustment. After the completion of over 50 performed test CT-FFR_ML_ analyses, the mean procedural time for the CT-FFR_ML_ analysis of our 88 enrolled patients was 23.9 ± 11.2 min. Besides a reduced processing time compared to conventional CT-FFR calculations, CT-FFR_ML_ demonstrates an excellent sensitivity (93%), specificity (94%), positive predictive value (93%) and negative predictive value (94%). 

### 3.5. Invasive Coronary Intervention

Revascularization therapy was conducted in 40 (45%) patients [PCI: 38 (95%); CABG: 2 (5%)]. In 39 (44%) patients, the hemodynamic severity was assessed invasively by either FFR [11 (13%)] or iwFR [28 (32%)]. During ICA 38 (43%) patients did not show any lesion with a diameter stenosis ≥50%, 35 (40%) revealed a one-vessel, 11 (13%) a two-vessel and four (5%) a three-vessel disease (Table 5).

### 3.6. Radiation Exposure

The mean total radiation exposure during cCTA was 617 ± 406 mGy*cm (Calcium-scoring: 44 ± 52 mGy*cm, cCTA: 551 ± 368 mGy*cm) with a mean contrast material use of 80.8 ± 15.4mL. In ICA the median of the radiation dose was 5648.0 mGy*cm and the mean amount of contrast material 158 mL (Table 3 and Table 5).

## 4. Discussion

In the present study, we demonstrated the potential effect of an on-site CT-FFR_ML_ machine learning algorithm in terms of clinical practicality and its diagnostic performance to determine the hemodynamic significance of coronary lesions. With the prototype software used in this study, 93% of our enrolled patients with suspected CAD or suspected progression of CAD who initially received CTA, were adequately classified. Using CT-FFR analysis, 55% of the patients enrolled could have potentially omitted further invasive testing and associated radiation exposure. Only 3% of patients had a CT-FFR_ML_ measurement of ≥0.8, despite the necessity of coronary revascularization.

In earlier studies, less than 60% of patients with suspected CAD and pathological non-invasive functional test results demonstrate a significant coronary obstruction during ICA [2,29]. However, other results might be possible if another stress test was used initially. In a more current study (MR-INFORM), it was shown that decisions in the diagnostic pathway in patients with a higher risk of a cardiovascular event can be guided as safely by cardiac magnetic resonance imaging (CMR) as they are by invasive FFR. Regarding the ISCHEMIA trial as well as the MR-INFORM study, always in light of the specific patient collective, it is clear that patients should be selected precisely to be referred to ICA [15,16,30]. This requires the integration of patient information by generating risk-stratification scores estimating the presence of obstructive CAD as well as non-invasive new imaging technics using self-improving machine learning software. With a higher sensitivity (95–99% [4,6]) compared to the current non-invasive functional testing methods and a high negative predictive value, cCTA helps reduce the number of patients without obstructive CAD during ICA [2,3]. The NXT-Trial study was able to reveal an improved specificity of cCTA when extended by CT-FFR [7]. Evaluating its additive value in clinical routine, the PLATFORM compared any form of non-invasive testing, including cCTA without CT-FFR, to cCTA plus CT-FFR and revealed 61% less referrals to ICA in the cCTA/CT-FFR group, resulting in significantly lower rates of unnecessary, non-obstructive ICAs. The real-world utility and safety of CT-FFR was consequently evaluated in the multicentre ADVANCE registry study [31]. Here, the disease management plan of two-thirds of patients in the cCTA plus CT-FFR group was changed compared to the patients in the core lab cCTA-only approach group. No death or myocardial infarction (MI) occurred in patients with a CT-FFR value of >0.80 within a 90 day follow-up.

The results of our retrospective study integrate into the outcomes of the leading international multi-centre ADVANCE registry and PLATFORM study, meeting the expectations of the “gatekeeper” function of CT-FFR. Similar to these studies, 48 (55%) out of 88 of our patients did not show any obstructive findings during ICA. By retrospectively adding the CT-FFR_ML_ diagnostics into the diagnostic pathway, we were able to show the additional value of combining morphological and functional information that could lead to a reduction in invasive procedures by 55%, as well as substantially lower incidence of finding no obstructive coronary disease during ICA.

Among a subgroup in the PLATFORM-study [18] who had invasive testing planned, 24 (12.4%) of 193 in the CT-FFR arm did not have hemodynamically significant lesions when assessed by ICA, compared to 137 (73.3%) in the usual care arm, a difference of 60.9%. In the CT-FFR arm, 17 (8.8%) had to be excluded due to poor image quality or inadequate acquisition. After the sole anatomical evaluation of the cCTA data in our study, 48 (55%) of 88 would have received ICA without the necessity of revascularization treatment, versus three (8%) of 40 patients after the analysis of the cCTA data by CT-FFR_ML_, a difference of 47%. PLATFORM compares the cCTA/CT-FFR group to the usual care group within the preselected “planned ICA” arm; therefore, all patients in the usual care group receive ICA and the effect of the integration of CT-FFR is even larger than in our study, since our hypothetical control group is already selected by cCTA. Similarly, we had to exclude 11 (8%) patients due to poor image quality or inadequate acquisition which might be a limitation of CT-FFR_ML_ or cCTA itself. Despite the retrospective study design, our study population was carefully selected. The number of patients with coronary anomalies (7, 5%) and subtotal or chronic total occlusions (13 and 10%) indicate the real-world profile of the patient collective. Other than in the original CT-FFR study NXT-Trial, patients with total occlusions were not included, with a predefined CT-FFR value of 0.50. This would have increased the number of true positives, and therefore improved the diagnostic performance of this method. Since CT-FFR analysis was virtually available for every patient in the ADVANCE study, how the downstream treatment of each patient would have been without CT-FFR could not be determined [31]. Since our study has a retrospective design, we were able to gather the respective information required to simulate the hypothetical CT-FFR_ML_ pathway and compare it to the actual diagnostic pathway that was chosen by the physician.

The series of diagnostic tools used by us, including the CAD consortium basic score, the Agatston score, the comprehensive CTA score and the CT-FFR analysis, reflects how the integration and combination of patient data leads to a precise risk stratification guiding a corresponding sophisticated therapeutic strategy. The discriminatory value of the CAD consortium basic pretest probability score is not displayed in the results of our study (AUC = 0.578, *p* = 0.3212) (Figure 4). The clinical score was previously evaluated in view of a diameter stenosis ≥50% in cCTA, not regarding revascularizing therapy [23]. Therefore, our population is biased, since it is a selection of patients who were referred to ICA after a positive cCTA. The means of the Agatston score showed a significant difference in the revascularized and not-revascularized group (*p* = 0.0467). However, the validity of this score is restricted due to a large standard deviation, since the amount of calcium in the coronary tree is just sufficiently able to predict the necessity of revascularization therapy (AUC = 0.631, *p* = 0.1448). Therefore, a high calcium score does not inevitably indicate myocardial ischemia; its prognostic accuracy is discussed, since it does not include non-calcified lesions and does not display if the amount of coronary calcium is accumulated in on spot or spread diffusely across the coronary tree [21]. The comprehensive CTA score offered a slightly better diagnostic accuracy (AUC = 0.633, *p* = 0.0158), which shows that including the typicity as well as the location of the lesion has an impact on the chosen therapy (Figure 4). The higher the comprehensive CTA score, the higher the risk of needing revascularization. However, the discriminatory value of this score from previous trials [22] could not be shown in this study due to the selection of our patient collective, patients who received both cCTA and ICA. Finally, the ischemia producing factor, the hemodynamic severity is quantified by the CT-FFR value and therefore provides a great discriminatory value (AUC = 0.958, *p* ≤ 0.0001) (Figure 4) to guide revascularization therapy.

Easy accessibility and the economical operation of the CT-FFR_ML_-software are essential for its integration into clinical practice [32,33]. The off-site CT-FFR software by Heartflow Inc. (1400 Seaport Blvd, Redwood City, CA 94063) has attained approval by the Food and Drug Administration in 2015, thus indicating the growing importance and utility of CT-FFR [7,34]. However, the CT-FFR software used in these studies was based on computational fluid dynamics. Thus, CTA data has to be transferred to external core laboratories and has to be analysed off-site, which takes several hours and implies great costs. With this setup the assessment is not accessible instantly and does not help in the clinical decision process [32]. In our study, we used a prototype software based on machine learning (cFFR, version 3.1, Siemens Healthineers, Forchheim, Germany) that allows an on-site computation on a regular workstation. Tesche et al. recently proved that the algorithm based on machine learning receives adequate results compared to the algorithm based on computational fluid dynamics, while significantly improving clinical practicability [20]. Our mean calculation time was 24 ± 11 min. An earlier publication by Renker et al. in 2014 demonstrated a processing and calculation time of 37.5 ± 13.8 min using an on-site prototype CT-FFR software which was based on a computational fluid dynamic algorithm [35]. The type of algorithm of the software, either CT-FFR based on machine learning (CT-FFR_ML_ or CT-FFR based on computational fluid dynamics (CT-FFR_CFD_), as well as the type of algorithm for the reconstruction of cCTA-data, seems to have an impact on the efficiency as well: iterative reconstruction in CT-FFR_ML_ lowers the post-processing speed compared to the filtered-back projection reconstruction of CT-FFR_CFD_ [36]. 

Using the data of the PLATFORM-study, Hlatky et al. analysed the costs and the Quality-of-Life when integrating CT-FFR_ML_ into the clinical practice. In the group of patients planned for ICA, the new diagnostic pathway lead to a 32% reduction (7.343 vs. 10.734 USD, *p* < 0.0001) in healthcare expenditures [37]. Standardized questionnaires as a part of this study revealed a significantly improved Quality-of-Life in the CT-FFR branch [37].

Although CT-FFR_ML_ is a very promising new non-invasive method in the disease management plan of patients with suspected obstructive CAD, there are a few caveats to consider. A total of 13% in the NXT-Trial, 8.8% in the PLATFORM study, and 8% of the patients in our study selected for CT-FFR analysis, had to be excluded because of inadequate image quality [7,18]. Furthermore, a “cut-off” value that allows a safe decision if the stenosis is obstructive or irrelevant has just been derived from the invasive FFR-Index (0.75–0.80) and has never been prospectively examined [38]. The result is a “grey-zone” of 0.7 to 0.9, which does not clearly indicate a normal or pathological CT-FFR_ML_ value [39]. Founded in the retrospective profile of our study, we exposed three (3%) false negative cases, patients who received stents but revealed a CT-FFR_ML_-value ≥ 0.80. Although single cases, two of the three false negatives revealed an iwFR value between 0.86 and 0.89 (grey-zone: 0.86–0.93 [40]) and one of these had a very high profile of risk factors. 

Our results furthermore support the importance of artificial intelligence whose rising capability and efficiency allows the development of medical software that instantly assists in clinical patient management. This therefore enables further “exploitation of the considerable richness of coronary CTA data” [32], leading to a more time and also a cost-efficient process. 

## 5. Limitations

First, the present study is limited due to its retrospective study design and its relatively small number of included patients. Furthermore, we do not have the respective invasive FFR- or iwFR-value as a reference to the CT-FFR_ML_ measurement in every patient [39 (44%) of 88 patients], since pressure-derived indices are only obtained if further information about the severity of the stenosis is required to determine the optimal treatment strategy for the patient. A hemodynamically significant lesion that can definitely be identified visually in ICA would not be assessed by an iwFR- or FFR-pressure wire. In these cases, the interventional cardiologist would proceed to a revascularization therapy immediately. A patient with a lesion that is clearly classified to be hemodynamically not flow-limiting visually would not undergo the risk of an iwFR- or FFR-measurement, which needs the introduction of a guidewire into the coronary artery and would therefore be referred to an optimal medical treatment plan or, e.g., a coronary computer tomographic angiography for a regular check-up. Limiting the results of our study is the number of patients (48 out of 136) we had to exclude due to technical reasons. In these patients, the impact of CT-FFR_ML_ cannot be demonstrated since their cCTA data was initially not suitable for the algorithm. However, it is questionable if these patients (CABG, subtotal or total occlusions, coronary anomalies or a stent in the vessel of interest) should be evaluated in clinical practice by a test that is preferably used in patients with an intermediate risk of obstructive CAD. The discriminatory value of the pretest probability and the comprehensive cCTA score, as in other studies, is not displayed due to the selection of our patient collective. However, it describes our patient population and underlines the potential value of CT-FFR_ML_ at this point of the diagnostic pathway, where patients have been preselected by cCTA. Furthermore, the reclassification in our study is simply guided by the single CT-FFR_ML_ value and does not consider a post CT-FFR_ML_ treatment plan by an experienced physician. 

## 6. Conclusions

We could demonstrate that adding CT-FFR_ML_ could have helped in the clinical decision process to potentially retain 55% of our patients from ICA, who might have only received a tailored medical therapy directly. In the future, developments in cCTA technology might add additional cardiac information. Due to its low invasiveness combined with decreased radiation exposure, cCTA might even have the potential to be a screening test to eliminate “silent myocardial ischemia” [41,42]. 

Therefore, CT-FFR_ML_ integrated into daily clinical routine can potentially spare unnecessary radiation exposure and a higher contrast dosage, as well as the costs and time of an invasive procedure. The CT-FFR_ML_ analysis software effectively differentiates between hemodynamic significant stenoses that needed further invasive diagnostics or even revascularization and stenosis that would have been classified as non-obstructive by ICA with a high sensitivity and specificity. As previous trials showed, CT-FFR_ML_ could be beneficial when integrated into daily clinical practice, since it seems to be associated with a markedly reduced rate of ICA showing no obstructive CAD.

## Figures and Tables

**Table 1:** Baseline characteristics (*n* = 88).

Demographics	Mean Value ± Standard Deviation or Frequency (%)
Age, mean ± SD (years)	63 ± 11
Male, no (%)	65 (74)
BMI, mean ± SD [kg/m^2^]	29 ± 5
**Cardiovascular risk factors**	
Hypertension *, no. (%)	69 (78)
Hyperlipidemia **, no. (%)	39 (44)
Diabetes mellitus, no. (%)	13 (15)
Smoker, no. (%)	34 (39)
Family history of CAD, no. (%)	19 (22)
**Angina type**	
Typical angina, no. (%)	19 (22)
Atypical angina, no. (%)	12 (14)
Non-cardiac chest pain, no. (%)	53 (60)
Pretest probability ***, mean ± SD (%)	25 ± 21
**Baseline medication**	
aspirin, no. (%)	24 (27)
P2Y_12_ inhibitor, no. (%)	3 (3)
statin, no. (%)	31 (35)
beta-blocker, no. (%)	32 (36)
calcium channel blocker (CCB), no. (%)	15 (17)
angiotensin-converting-enzyme inhibitor or AT_1_ receptor blocker (ARB), no. (%)	46 (52)
nitrates, no. (%)	1 (1)
**Baseline blood values**	
Cholesterol, mean ± SD [mg/dl]	195 ± 59
High-density lipoprotein, mean ± SD [mg/dl]	51 ± 20
Low-density lipoprotein, mean ± SD [mg/dl]	117 ± 45
Triglycerides, mean ± SD [mg/dl]	213 ± 213
Hemoglobin A1c, mean ± SD [%]	6 ± 1
Creatine kinase, mean ± SD [U/l]	188 ± 254
Creatine kinase muscle-brain type, mean ± SD [U/l]	45 ± 86
High sensitive Troponin-I > 0.045, no. (%)	4 (5)

Unless otherwise specified, data are numbers of patients with percentage in parentheses. Data are mean ± standard deviation (SD). * Defined as blood pressure >140 mmHg systolic, >90 mmHg diastolic, or use of an antihypertensive medication. ** Defined as a total cholesterol level of >200 mg/dL or use of lipid lowering medication. *** Calculated with the CAD consortium basic score [23]. CAD = coronary artery disease, BMI = body mass index, SD = standard deviation.

**Table 2:** Clinical and CT-derived scores and CT-FFR_ML_.

	Total	Not Revascularized, (Median)	Revascularized, (Median)	OR	95% CI	AUC	*p*-Value
**Pre-test probability (%)**	88	14.00	20.50	1.010	0.990–1.032	0.578	0.3212
**Agatston score**	79	207.20	429.20	1.055	1.000–1.001	0.631	0.1448
**Comprehensive CTA score**	88	7.16	10.79	1.110	1.020–1.208	0.633	0.0158
	**Total**	**Not revascularized, (Mean ± SD)**	**Revascularized, (Mean ± SD)**	**OR**	**95% CI**	**AUC**	***p*–Value**
**CT-FFR_ML_**	88	0.89 ± 0.08	0.58 ± 0.15	0.138	0.062–0.309	0.958	<0.0001

UC = area under the curve, OR = odds ratio, CI = confidence interval, SD = standard deviation.

**Table 3:** Findings of cCTA (*n* = 88).

Coronary Computed Tomography Angiography—Lesion Location	
Left main truncus *, no. (%)	0 (0)
Left anterior descending *, no. (%)	60 (68)
Left circumflex artery *, no. (%)	11 (13)
Right coronary artery *, no. (%)	17 (19)
**Stenosis grade in cCTA [26]**	
Moderate: 50–69% diameter stenosis, no. (%)	35 (40)
Severe: 70–99% diameter stenosis, no. (%)	53 (60)
Occluded, no (%)	0 (0)
**Risk stratification and Image Quality**	
Agatston score *, mean ± SD	553 ± 651
Agatston score *, Range	6–3264
Agatston score *, no. of patients >400 (%)	33 (38)
Comprehensive CTA score **, mean ± SD	9.72 ± 5.47
Image quality ***, mean ± SD	4.2 ± 0.8
**Radiation Exposure**	
Mean ± SD [mGy*cm]	617 ± 406
Median [mGy*cm]	553
Contrast agent ± SD [mL]	80.8 ± 15.4

Unless otherwise specified, data are numbers of patients with percentage in parentheses. Data are mean ± standard deviation (SD). * Agatston score was obtained in 78 (89%) patients. ** Calculated in 88 patients. *** Evaluation by two observers using a 5-point Likert scale: from 1 = non-diagnostic to 5 = excellent. SD = standard deviation, LVEF = left ventricular ejection fraction.

**Table 4:** Reclassification.

	CT-FFR_ML_		Total no. (%)
	≤0.80	>0.80	
obstructive CAD	37 (42.0%)	3 (3.4%)	40 (45.5%)
no obstructive CAD	3 (3.4%)	45 (51.1%)	48 (54.5%)
total no. (%)	40 (45.5%)	48 (54.5%)	88 (100%)

CAD = coronary artery disease, CT-FFR_ML_ = Machine-learning-based CT-derived fractional flow reserve.

**Table 5:** Findings of ICA (*n* = 88).

Invasive Coronary Angiography	
Contrast agent ± SD [mL]	158 ± 106
X-ray time ± SD [min]	7.4 ± 6.2
Dose area product ± SD [cGy*cm^2^]	11,900 ± 21,080
One-vessel disease, no. (%)	35 (40)
Two-vessel disease, no. (%)	11 (13)
Three-vessel disease, no. (%)	4 (5)

Unless otherwise specified, data are numbers of patients, with percentage in parentheses. Data are mean ± standard deviation (SD) or frequency. CAD = coronary artery disease, SD = standard deviation.

## Abbreviations

CAD	coronary artery disease
CCS	chronic coronary syndrome
cCTA	coronary computed tomography angiography
CT-FFR_CFD_	CT-derived fractional flow reserve based on computational fluid dynamics
CT-FFR_ML_	CT-derived fractional flow reserve based on machine learning
FFR	fractional flow reserve
ICA	invasive coronary angiography
iwFR	instantaneous wave-free ratio
OMT	optima medical therapy
